# Impact of psychological distress on work productivity among confectionery workers

**DOI:** 10.1192/j.eurpsy.2026.11657

**Published:** 2026-07-31

**Authors:** H. Daoud, I. Sellami, A. Feki, S. B. C. Ben Chaaben, M. Hajjaji, M. L. Masmoudi, K. Jmal Hammami

**Affiliations:** 1 Department of occupational medicine, Hedi Chaker Hospital; 2 LR/18/ES-28, Sfax university; 3 Department of rheumatology, Hedi Chaker Hospital; 4 Department of occupational medicine, Occupational Medicine, Sfax, Tunisia

## Abstract

**Introduction:**

The confectionery sector is characterized by physically demanding and stressful working conditions, which may impact employees mental health and productivity.

**Objectives:**

This study aimed to examine the association between mental health disorders and work productivity loss among confectionery workers.

**Methods:**

A cross-sectional survey was conducted among workers in a private confectionery in Sfax. Data were collected between December 2022 and July 2023 using a pre-established questionnaire. Mental health assessment was performed using the 21-item Depression, Anxiety, and Stress Questionnaire (DASS21). Work productivity was assessed using the Work Productivity and Activity Impairment (WPAI) questionnaire.

**Results:**

Our study included 200 participants. Mean age was 32.33±8.83 years. Women represented 61%. Mean scores were 3.71±4.11 for depression, 3.98±3.60 for anxiety, and 4.91±4.85 for stress. The mean absenteeism was 4.43 ±13.50%, while presenteeism showed a mean of 19.40 ±30.71%. Mean total work productivity loss was 22.26 ± 31.85%. Bivariate analysis indicated that higher levels of depression (r = 0.40, p < 0.001), anxiety (r = 0.44, p < 0.001), and stress (r = 0.33, p < 0.001) were significantly associated with greater overall work productivity loss.

**Conclusions:**

Higher psychological distress is associated with greater work productivity loss, highlighting the importance of addressing psychological distress in the workplace to improve employee well-being and overall productivity.

**Disclosure of Interest:**

None Declared

